# Mitochondrial dysfunction in ARDS: unraveling the regulatory networks and therapeutic opportunities

**DOI:** 10.3389/fimmu.2026.1899718

**Published:** 2026-07-29

**Authors:** Yu-Huan Li, Miao Wang, Ke Wan, Jun Li, Xiao-Feng Li

**Affiliations:** 1 Department of Tuberculosis, Anhui Chest Hospital, Hefei, China; 2 Inflammation and Immune Mediated Disease Laboratory of Anhui Province; The Key Laboratory of Anti-inflammatory and Immune Medicines, Ministry of Education; School of Pharmacy, Anhui Medical University, Hefei, China; 3 Department of Orthopedics, The Second Affiliated Hospital of Anhui Medical University, Hefei, China

**Keywords:** acute respiratory distress syndrome, glycolysis, metabolic reprogramming, mitochondrial dysfunction, mtDNA, therapeutic targeting

## Abstract

Acute respiratory distress syndrome (ARDS) is a life-threatening condition with high mortality and limited effective pharmacotherapies. Accumulating evidence has established mitochondrial dysfunction as a central pathogenic hub in ARDS. Injured mitochondria exhibit excessive reactive oxygen species production, impaired mitophagy, aberrant dynamics (predominantly Drp1-mediated fission), reduced biogenesis, and release of mitochondrial DNA as a damage-associated molecular pattern. These alterations trigger inflammatory cascades via the cGAS/STING and NLRP3 pathways, while simultaneously driving a metabolic shift from oxidative phosphorylation to aerobic glycolysis, the Warburg effect. Key glycolytic enzymes, including PKM2, PDK4, GAPDH, and PGK1, reinforce mitochondrial damage through lactate production and HIF-1α stabilization, creating a vicious cycle. Notably, PFKFB3 exhibits cell-type-specific duality, exerting protective effects in alveolar epithelial cells while promoting NETosis in neutrophils. Distinct cell types in the lung, alveolar macrophages, neutrophils, alveolar epithelial cells, and pulmonary endothelial cells exhibit unique mitochondrial and metabolic alterations that collectively perpetuate injury and impair repair. Therapeutically, mitochondria-targeted agents (MitoQ, MOTS-c), modulators of mitochondrial dynamics (baicalein, hydrogen), inhibitors of glycolytic enzymes (PFKFB3, PKM2, PDK4), natural compounds (1-octyl itaconate, shikonin, scutellarin), and mesenchymal stromal cell-mediated mitochondrial transfer have shown promise in preclinical models. This review synthesizes current understanding of the regulatory mechanisms linking mitochondrial dysfunction and metabolic reprogramming in ARDS, discusses cell-type-specific contributions, and highlights emerging therapeutic strategies. Targeting mitochondrial homeostasis and the associated glycolytic shift may offer a transformative approach to ARDS treatment, though challenges related to cell specificity, safety, and clinical translation remain.

## 1 Introduction

Acute lung injury (ALI) and its more severe form, acute respiratory distress syndrome (ARDS) are life-threatening clinical conditions with an incidence ranging from 10.1 to 86.2 per 100,000 person-years and mortality rates reaching 30-40%, exceeding 35% in severe cases (1, 2). ARDS arises from diverse etiologies, including sepsis, bacterial pneumonia, multiple trauma, aspiration pneumonia, and chemical lung injury (3, 4). Despite significant advances in mechanical ventilation and pharmacologic interventions, mortality remains alarmingly high. Current treatment strategies are largely supportive, primarily relying on lung-protective ventilation and restrictive fluid management, with a notable absence of effective specific pharmacotherapies (5–7). This therapeutic gap underscores the urgent need for novel interventions targeting the underlying pathophysiological mechanisms of ARDS.

Although ARDS arises from heterogeneous etiologies, it is unified by common pathological features, including dysregulated systemic inflammation, oxidative stress, increased endothelial and epithelial permeability, and diffuse alveolar damage (4, 5). These changes collectively result in alveolar edema, fibrosis, necrosis, and the deposition of proteinaceous exudates, severely compromising respiratory function. The underlying pathophysiology is driven by an imbalance in the inflammatory response, in which excessive immune activation triggers a deleterious inflammatory cascade. Clinically, ARDS is typically divided into three overlapping phases: the exudative phase, characterized by alveolar-capillary barrier disruption, proteinaceous edema fluid infiltration, and neutrophil-predominant inflammation (8); the proliferative phase, defined by tissue repair and organization; and the fibrotic phase, during which pulmonary fibrosis may develop, contributing to long-term respiratory dysfunction (9).

A prominent pathological hallmark is the marked accumulation of activated immune cells, which play central roles in disease progression. Key cellular contributors include alveolar macrophages, which undergo M1 polarization and drive pro-inflammatory responses; neutrophils, which mediate tissue damage through the formation of neutrophil extracellular traps (NETs); alveolar epithelial cells (both type I and type II), whose integrity is critical for barrier function and repair; and vascular endothelial cells, which regulate vascular permeability and leukocyte trafficking (10, 11).

**Figure 1:**
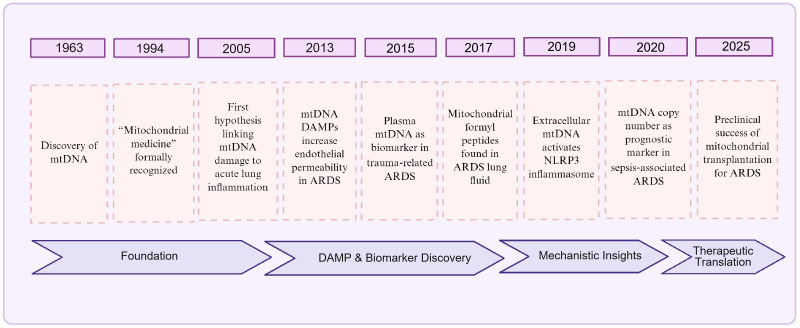
The research progress of mitochondria in ARDS. The research on mitochondria in ARDS from 1963 to 2025 is divided into four stages: Preliminary Discovery (1963–2005), Biomarker Discovery (2006–2017), Mechanism Exploration (2018–2020), and Therapeutic Translation (2021 to Present).

Mitochondria are the primary energy-producing organelles and central hubs for signaling, redox regulation, and programmed cell death (12, 13). Accumulating evidence has established mitochondrial dysfunction as a critical determinant of ARDS pathogenesis. The temporal evolution of mitochondrial research in ARDS is summarized in **Figure 1**. In the inflammatory microenvironment of ARDS, mitochondria undergo profound functional and structural alterations including excessive reactive oxygen species (ROS) production, impaired mitophagy, aberrant mitochondrial dynamics, reduced biogenesis, and release of mitochondrial DNA (mtDNA) as a damage-associated molecular pattern (DAMP), that collectively drive disease progression (1, 14, 15).

The designation of mitochondrial dysfunction as a “central hub” in ARDS reflects its unique upstream and integrative position in pathophysiology. Unlike downstream cytokine cascades or isolated cell-type-specific effects, mitochondrial injury directly couples diverse initial insults to the NLRP3 inflammasome (16) and cGAS-STING pathways (12), while simultaneously driving barrier disruption (10), metabolic reprogramming (17) and inflammatory cell death (18) across all major pulmonary cell types. This convergence establishes mitochondrial homeostasis as a nodal point controlling the vicious cycle of inflammation, barrier failure, and metabolic dysregulation (14). The detailed mechanistic evidence supporting this central role, encompassing oxidative stress, mitophagy, dynamics, biogenesis, mtDNA signaling, and metabolic reprogramming, is elaborated in Section 2.

Based on this framework, this review aims to summarize current knowledge on the regulatory mechanisms of mitochondrial dysfunction in ARDS and explore the therapeutic potential of targeting mitochondrial homeostasis in this condition.

## 2 Mitochondrial dysfunction in ARDS: mechanistic landscape

**Figure 2:**
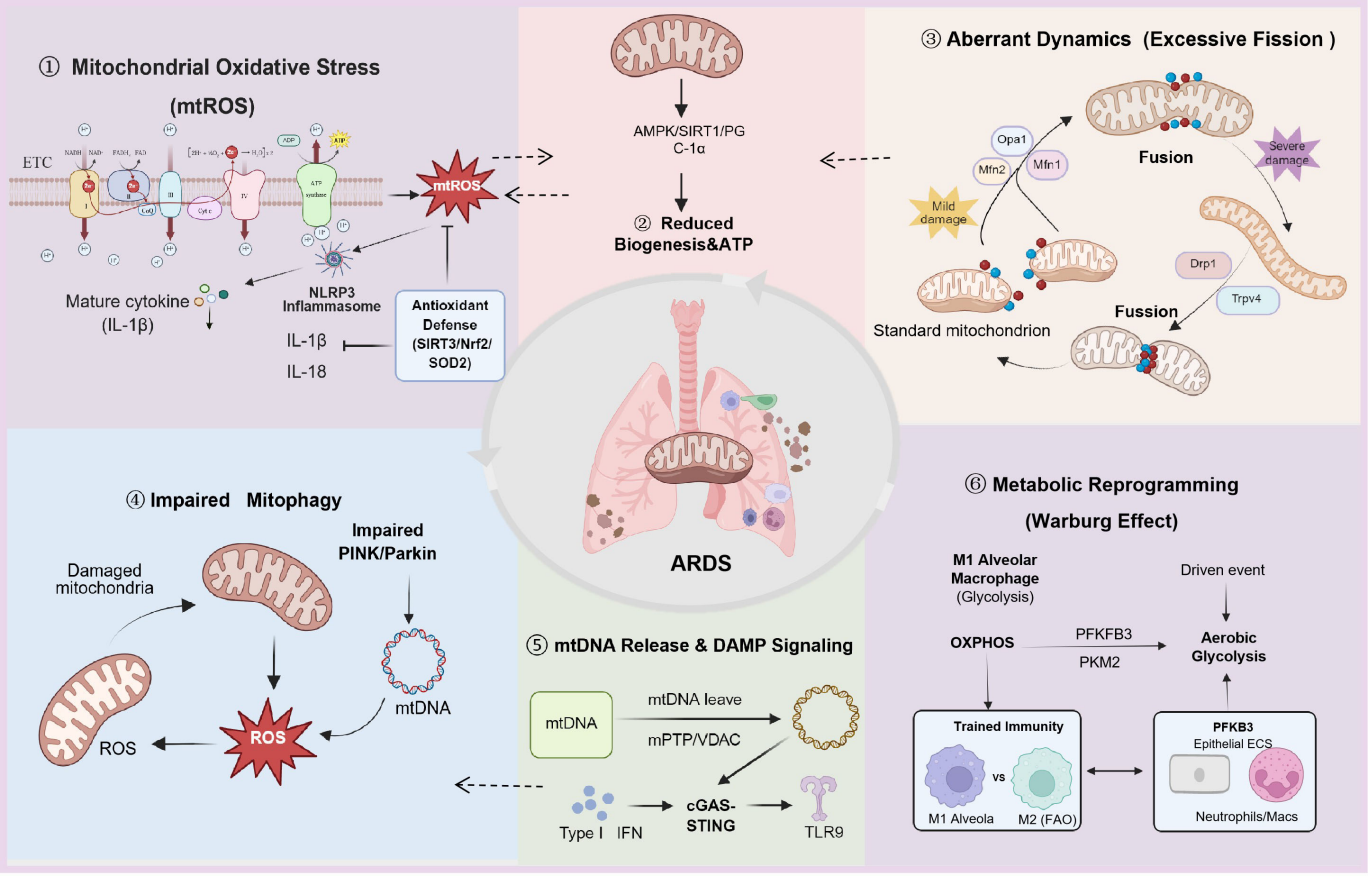
Schematic overview of mitochondrial dysfunction pathways mediating ARDS. Six interrelated mitochondrial pathological cascades synergistically promote pulmonary inflammatory injury in ARDS: (1) Mitochondrial oxidative stress: ETC dysfunction overproduces mtROS, which inhibits SIRT3/Nrf2/SOD2 antioxidant signaling and activates NLRP3 inflammasome to secrete mature IL-1β/IL-18. (2) Reduced biogenesis and ATP depletion: Suppressed AMPK/SIRT1/PGC-1α signaling blunts mitochondrial biogenesis and ATP generation. (3) Excessive mitochondrial fission: Severe damage disrupts Mfn1/Mfn2/Opa1-mediated fusion and triggers Drp1/TRP4-dependent aberrant fission. (4) Impaired mitophagy: Defective PINK/Parkin clearance of damaged mitochondria sustains ROS overaccumulation. (5) mtDNA release and DAMP signaling: mtDNA leaks through mPTP/VDAC, activating cGAS-STING-type I IFN and TLR9 inflammatory cascades. (6) Warburg-type metabolic reprogramming: Downregulated OXPHOS upregulates PFKFB3/PKM2 to drive aerobic glycolysis in alveolar macrophages, epithelial endothelial cells and neutrophils, shifting M1/M2 macrophage polarization and boosting trained immunity. All pathways form positive feedback loops to exacerbate alveolar damage and ARDS progression.

Mitochondrial dysfunction in ARDS is not a single isolated defect but rather a network of interconnected alterations that collectively drive pathogenesis. These include excessive reactive oxygen species production, impaired mitophagy, aberrant mitochondrial dynamics, reduced biogenesis, mtDNA-mediated inflammation, and a metabolic switch toward aerobic glycolysis. These processes are not independent; rather, they reinforce one another through feed-forward loops that sustain and amplify lung injury. **Figure 2** provides an integrative overview of these major mitochondrial pathways and illustrates their mechanistic interconnections and pathological convergence in ARDS. The following subsections discuss each component in detail.

### 2.1 Mitochondrial oxidative stress and antioxidant defense

Mitochondria are the primary source of intracellular reactive oxygen species (ROS), with complexes I and III of the electron transport chain (ETC) serving as the major sites of superoxide production (19, 20). In ARDS, excessive mitochondrial ROS (mtROS) production plays a central role in driving inflammation, barrier disruption, and cell death. Under inflammatory conditions, stimuli such as lipopolysaccharide (LPS), pro-inflammatory cytokines, and hypoxia activate mtROS production through multiple mechanisms, with ETC dysfunction leading to electron leakage and increased superoxide generation (21). Elevated mtROS levels have been consistently observed in alveolar macrophages, neutrophils, endothelial cells, and epithelial cells during ARDS (16, 22).

A key downstream consequence of mtROS elevation is activation of the NLRP3 inflammasome. Mechanistically, mtROS triggers NLRP3 inflammasome assembly via thioredoxin-interacting protein (TXNIP) dissociation and mitochondrial dysfunction (16), leading to caspase-1 activation and subsequent cleavage of pro-IL-1β and pro-IL-18 into their mature forms, thereby amplifying inflammation. The pathological relevance of this axis is underscored by findings that cigarette smoke priming for Pseudomonas aeruginosa-induced acute lung injury involves NLRP3 inflammasome activation (23).

To counteract mtROS, cells possess a sophisticated antioxidant defense system. Superoxide dismutase 2 (SOD2), localized in the mitochondrial matrix, catalyzes superoxide dismutation to hydrogen peroxide, which is subsequently detoxified by glutathione peroxidase and catalase. Sirtuin 3 (SIRT3), a NAD^+^-dependent mitochondrial deacetylase, critically activates SOD2 and other antioxidant enzymes through deacetylation (16, 22); its downregulation under oxidative stress contributes to increased mtROS levels. The nuclear factor erythroid 2-related factor 2 (Nrf2) pathway serves as a master regulator of antioxidant responses, inducing heme oxygenase-1 (HO-1) expression. Notably, mitoquinone mesylate (MitoQ), a mitochondria-targeted antioxidant, protects against endothelial barrier hyperpermeability via an Nrf2-dependent mechanism (22), underscoring the central role of oxidative stress in sustaining inflammatory injury. In addition to these defense mechanisms, mitochondrial DNA (mtDNA) is particularly vulnerable to oxidative damage due to its proximity to ETC-generated ROS and lack of protective histones; oxidative damage to lung mtDNA is a key contributor to chemical lung injury (24), and damaged mtDNA released into the cytoplasm or extracellular space acts as a damage-associated molecular pattern (DAMP) to activate inflammatory signaling. Notably, mitochondrial ROS are not exclusively pathogenic; at low to moderate concentrations, they function as essential second messengers that modulate redox-sensitive signaling pathways, including those involved in hypoxic adaptation and innate immune priming (25). The pathological outcomes of mitochondrial ROS in ARDS are therefore highly dose- and context-dependent, with excessive and sustained production overwhelming endogenous antioxidant defenses and triggering tissue injury.

### 2.2 Mitophagy: quality control gone awry

Mitophagy is a selective form of autophagy that eliminates damaged or dysfunctional mitochondria, thereby maintaining mitochondrial quality control (26, 27). In ARDS, impaired mitophagy contributes to the accumulation of damaged mitochondria, exacerbating oxidative stress and inflammation (28–30). The PINK1/Parkin pathway is the best-characterized mitophagy mechanism: upon mitochondrial depolarization, PINK1 accumulates on the outer mitochondrial membrane, recruiting Parkin from the cytosol, which then ubiquitinates outer membrane proteins to promote autophagosome formation and mitochondrial degradation (31). In ARDS, this pathway is often dysregulated. HDAC3 deficiency protects against acute lung injury by preserving mitochondrial quality control (4), suggesting that enhancing mitophagy may be protective. Conversely, MitoQ ameliorates EtOH-LPS induced lung injury by inhibiting mitophagy and NLRP3 inflammasome activation (16), indicating that excessive mitophagy, rather than mitophagy per se, may be detrimental in certain contexts and that the therapeutic outcome depends on the magnitude and timing of mitophagic flux.

Alternative mitophagy pathways involve receptors such as BNIP3, NIX, and FUNDC1, which directly bind LC3 on autophagosomes. Hypoxia-inducible factor 1α (HIF-1α) induces BNIP3 and NIX expression, linking hypoxia to mitophagy. In ARDS, these pathways may be activated under hypoxic conditions, potentially serving protective or detrimental functions depending on context. Thus, the balance of mitophagic flux critically determines whether mitochondrial quality control exerts protective or detrimental effects in ARDS.

In addition to the pathways described above, the therapeutic implications of modulating mitophagy are heavily influenced by disease context and timing. While excessive or impaired mitophagy contributes to epithelial cell death and sustained inflammation in late-stage ARDS, basal mitophagy serves as an indispensable quality-control mechanism. Under physiological conditions or during early-stage injury, the selective removal of damaged mitochondria limits ROS amplification and prevents the release of pro-inflammatory mtDNA, thereby exerting a cytoprotective effect (32). This dual role highlights that therapeutic strategies targeting mitophagy must be precisely timed to preserve its homeostatic function while curbing its pathological overactivation.

### 2.3 Mitochondrial dynamics: fission and fusion imbalance

Mitochondrial dynamics, encompassing fusion and fission processes, are essential for maintaining mitochondrial function and cellular homeostasis; their imbalance critically contributes to ARDS pathogenesis through multiple mechanisms. Mitochondrial fission is primarily mediated by dynamin-related protein 1 (Drp1), and excessive Drp1-mediated fission has been observed in various cell types during ARDS. Activation of the mechanosensitive Ca²^+^ channel TRPV4 induces endothelial barrier permeability via disruption of mitochondrial bioenergetics (10), while inflammatory lung injury is associated with endothelial cell mitochondrial fission and requires RhoA nitration and cytoskeletal remodeling (12). Conversely, hydrogen alleviates impaired lung epithelial barrier by inhibiting Drp1-mediated fission through the Trx1 pathway (4), and baicalein suppresses LPS-induced lung injury by regulating Drp1-dependent fission in macrophages (15). Coral calcium hydride promotes peripheral mitochondrial division via activation of the Trx2/Myo19/Drp1 pathway (31). Notably, inhibition of STING-induced mitochondrial Drp1/N-GSDMD-mediated mtDNA release alleviates sepsis-induced lung injury (12), while 1-octyl itaconate ameliorates alveolar macrophage pyroptosis by rescuing mitochondrial dysfunction and suppressing the cGAS/STING pathway (1), demonstrating that mitochondrial dynamics and cGAS/STING signaling are interconnected in the regulation of pyroptosis.

In contrast, mitochondrial fusion is regulated by mitofusin 1 (MFN1), mitofusin 2 (MFN2), and optic atrophy protein 1 (OPA1). Beyond its role in fusion, MFN2 also regulates platelet mitochondrial function (6), and pyruvate kinase M2 (PKM2) modulates mitochondrial dynamics alongside epithelial-mesenchymal transition (EMT) in alveolar epithelial cells during sepsis-associated pulmonary fibrosis (8), highlighting that metabolic enzymes influence mitochondrial morphology. The balance between fission and fusion is critical, as excessive fission or impaired fusion both contribute to mitochondrial fragmentation and dysfunction in ARDS.

Beyond these mechanistic observations, mitochondrial dynamics, including fission and fusion, exhibit a dichotomous role in ARDS. Transient fission is a requisite for mitophagy and facilitates the distribution of mitochondrial metabolites during cellular stress responses, whereas excessive fission driven by Drp1 leads to mitochondrial fragmentation and dysfunction (33). The net effect on alveolar epithelial and endothelial integrity depends on the balance between adaptive remodeling and maladaptive fragmentation. The fission-fusion equilibrium is therefore a key determinant of mitochondrial homeostasis in ARDS.

### 2.4 Mitochondrial biogenesis: failure of regenerative capacity

Mitochondrial biogenesis is the process by which cells generate new mitochondria, essential for maintaining mitochondrial mass and function (34). Peroxisome proliferator-activated receptor gamma coactivator 1-alpha (PGC-1α) serves as the master regulator of this process, coordinating the expression of nuclear-encoded mitochondrial genes. The AMPK (AMP-activated protein kinase)/SIRT1 (sirtuin 1)/PGC-1α pathway plays a critical role: AMPK activation enhances NAD^+^ levels, activating SIRT1, which deacetylates and activates PGC-1α (24). In ARDS, this pathway is often suppressed, leading to reduced mitochondrial biogenesis and impaired cellular energetics. 17β-estradiol ameliorates LPS-induced acute lung injury via mediating mitochondrial biogenesis and function (14), suggesting that hormonal regulation influences this process.

S1PR3 inhibition protects against LPS-induced ARDS by inhibiting NF-κB and improving mitochondrial oxidative phosphorylation (11), indicating that modulating signaling pathways can enhance mitochondrial function. Additionally, mesenchymal stromal cell extracellular vesicles rescue mitochondrial dysfunction and improve barrier integrity in clinically relevant models of ARDS (34), potentially through promoting mitochondrial biogenesis in recipient cells. PGC-1α-mediated biogenesis is therefore essential for restoring mitochondrial mass and energetic capacity in the injured lung.

### 2.5 Mitochondrial DNA as a damage-associated molecular pattern

Mitochondrial DNA (mtDNA) shares structural similarities with bacterial DNA and acts as a potent damage-associated molecular pattern (DAMP) when released into the cytoplasm or extracellular space (35). In ARDS, mtDNA release from damaged mitochondria triggers inflammatory responses through multiple pathways. Oxidized DNA fragments exit mitochondria via mPTP- and VDAC-dependent channels to activate NLRP3 inflammasome and interferon signaling (3); this release is often triggered by oxidative stress, calcium overload, and mitochondrial depolarization. Inhibition of STING-induced mitochondrial Drp1/N-GSDMD-mediated mtDNA release alleviates sepsis-induced lung injury (12), demonstrating the importance of this pathway.

Once released, mtDNA activates pattern recognition receptors including Toll-like receptor 9 (TLR9) and the cGAS-STING pathway, leading to type I interferon production and inflammatory cytokine expression. 1-Octyl itaconate ameliorates alveolar macrophage pyroptosis by rescuing mitochondrial dysfunction and suppressing the cGAS/STING pathway (1), highlighting the therapeutic potential of targeting this axis. Moreover, mitochondrial DAMPs exacerbate lung fluid imbalance via the formyl peptide receptor-1 signaling pathway (35), demonstrating multiple pathways by which mtDNA promotes injury. Clinically, whole-blood mtDNA copies are associated with the prognosis of ARDS after sepsis (26), indicating that mtDNA levels may serve as a prognostic biomarker. mtDNA thus functions as a critical DAMP that links mitochondrial damage to systemic inflammatory activation.

### 2.6 Metabolic reprogramming: the Warburg effect and its interplay with mitochondrial dysfunction

**Figure 3:**
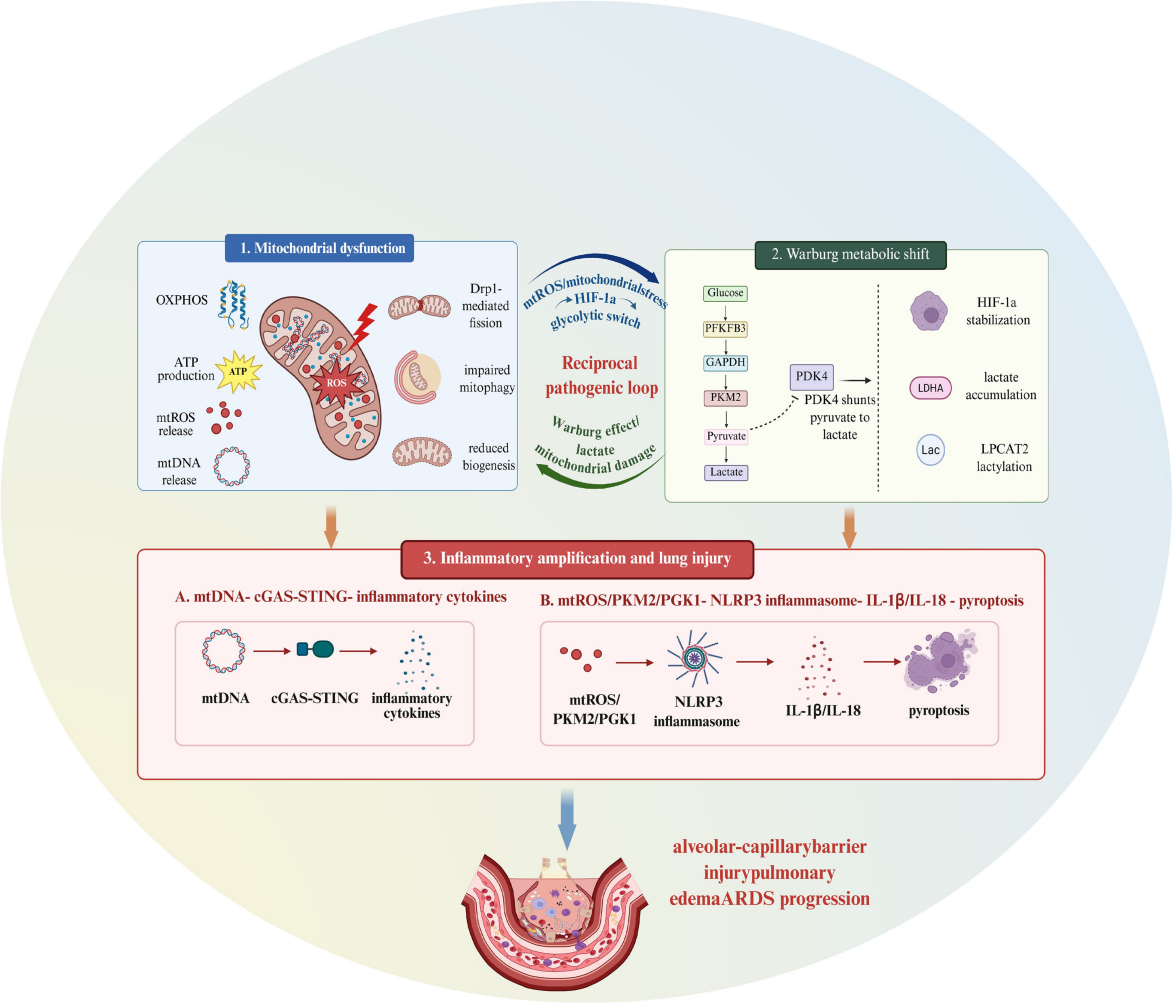
Schematic of the reciprocal pathogenic loop between mitochondrial dysfunction and Warburg metabolic shift driving ARDS. Mitochondrial damage induces mtROS/mtDNA release and HIF-1α-dependent glycolytic reprogramming; enhanced glycolysis reciprocally aggravates mitochondrial impairment. This vicious cycle activates two inflammatory axes: mtDNA-cGAS-STING cytokine signaling and mtROS/PKM2-triggered NLRP3 inflammasome-pyroptosis. Combined inflammation disrupts the alveolar-capillary barrier, causes pulmonary edema, and promotes ARDS progression.

ARDS typically features a shift from oxidative phosphorylation (OXPHOS) to aerobic glycolysis, actively driving inflammation and injury (17, 18, 36) (**Figure 3**). This metabolic switch is not merely an epiphenomenon but a critical pathogenic mechanism that reinforces mitochondrial damage and perpetuates the inflammatory response.

Several rate**-**limiting glycolytic enzymes have been identified as critical regulators, with roles that are markedly cell-type- and context-dependent. 6-phosphofructo-2-kinase/fructose-2,6-bisphosphatase 3 (PFKFB3) controls glycolytic flux; in neutrophils it promotes NET formation (37), and the related isoform PFKFB2-driven glycolysis promotes dendritic cell maturation (38). Interestingly, HIF-1α-dependent induction of alveolar epithelial PFKFB3 dampens ALI (39), revealing a striking cell-type-specific duality. PKM2 is targeted by Platycodin D3 to inhibit the PKM2-NLRP3 axis (40) and also modulates mitochondrial dynamics and epithelial-mesenchymal transition (EMT) (8). Pyruvate dehydrogenase kinase 4 (PDK4) inactivates pyruvate dehydrogenase, shunting pyruvate to lactate; PDK4-driven lactate accumulation facilitates lysophosphatidylcholine acyltransferase 2 (LPCAT2) lactylation, a novel post-translational modification that exacerbates sepsis-induced ALI (41). Glyceraldehyde-3-phosphate dehydrogenase (GAPDH) is covalently targeted by Pudilan formula and baicalein, suppressing the macrophage Warburg effect to alleviate ALI (17). Phosphoglycerate kinase 1 (PGK1) promotes M1 macrophage polarization and induces pyroptosis via NLRP3 regulation (18). Finally, LDHA is suppressed by growth differentiation factor 15 (GDF15), which attenuates sepsis-induced ALI through the HIF-1α/lactate dehydrogenase A (LDHA) pathway (42); similarly, scutellarin suppresses HIF-1α-mediated Warburg effect and pyroptosis in macrophages (43).

Beyond the actions of individual enzymes, the net balance between glycolysis and mitochondrial oxidative metabolism dictates immune cell polarization. M1 macrophages rely on aerobic glycolysis, while M2 macrophages depend on fatty acid oxidation (FAO). Carnitine palmitoyltransferase 1A (CPT1A)-mediated FAO promotes IL-10 production and M2 polarization, thereby ameliorating ALI (44). Protein 4.1R regulates M1 macrophage polarization via glycolysis, a mechanism that alleviates sepsis-induced liver injury and is likely relevant to the lung (45). The interplay between glycolytic reprogramming and NLRP3 inflammasome activation has been established as a critical determinant of ALI/ARDS progression (36).

Notably, not all glycolytic shifts are detrimental. Mitochondrial open reading frame of the 12S rRNA-c (MOTS-c), a mitochondrial-derived peptide, promotes glycolysis via the AMPK-HIF-1α-PFKFB3 pathway to ameliorate cardiopulmonary bypass (CPB)-induced lung injury (46). Trained immunity attenuates acute lung injury by activating alveolar macrophages through AKT2-PDK1 axis-mediated metabolic reprogramming (47). These examples suggest that controlled metabolic modulation can be harnessed therapeutically. Such context-dependent outcomes underscore that mitochondrial integrity, rather than glycolysis per se, ultimately determines cellular fate, a notion that forms the foundation of the therapeutic rationale elaborated in Section 4. The major mitochondrial pathway alterations discussed above and their corresponding therapeutic rationales are summarized in **Table 1**.

**Table 1:** Overview of key mitochondrial pathway alterations in ARDS and corresponding therapeutic rationales.

Mitochondrial pathway	Pathological alteration in ARDS	Key effector molecules	Therapeutic rationale	Representative agents	Refs.
Oxidative Stress	mtROS overproduction → NLRP3 inflammasome activation; endothelial barrier disruption	ETC complex I/III, SOD2, SIRT3, Nrf2	Scavenge mtROS; enhance endogenous antioxidant defenses	MitoQ	(16, 22, 25)
Mitophagy	Impaired clearance of damaged mitochondria → accumulation of dysfunctional organelles	PINK1, Parkin, BNIP3, NIX, FUNDC1, LC3	Restore mitochondrial quality control; enhance mitophagy	Melatonin	(27–30, 32)
Mitochondrial Dynamics	Excessive Drp1-mediated fission → fragmentation; impaired fusion	DRP1, MFN1, MFN2, OPA1, RhoA, TRPV4	Inhibit excessive fission; restore fission/fusion balance	Baicalein, H_2_, Coral calcium hydride	(4, 10, 12, 15, 31)
Mitochondrial Biogenesis	Suppressed PGC-1α pathway → reduced mitochondrial mass and function	PGC-1α, AMPK, SIRT1	Activate PGC-1α pathway; promote biogenesis	17β-Estradiol, S1PR3 inhibitors, MSC-EVs	(11, 14, 33)
mtDNA-Mediated Inflammation	mtDNA release into cytosol/extracellular space → cGAS/STING and TLR9 activation	cGAS, STING, TLR9, NLRP3, GSDMD	Inhibit mtDNA release or downstream signaling	1-Octyl itaconate, STING inhibitors (H-151)	(1, 3, 12)
Metabolic Reprogramming	Shift from OXPHOS to aerobic glycolysis (Warburg effect); FAO suppression	PFKFB3, PKM2, PDK4, GAPDH, PGK1, LDHA, HIF-1α	Inhibit glycolytic enzymes; restore OXPHOS; promote FAO	PFKFB3 inhibitors, PKM2 inhibitors, PDK4 inhibitors, GDF15, CPT1A modulators	(17, 18, 35–46)

Note: →indicates a downstream pathological consequence.

## 3 Cell-type specific roles of mitochondrial dysfunction in ARDS

**Figure 4:**
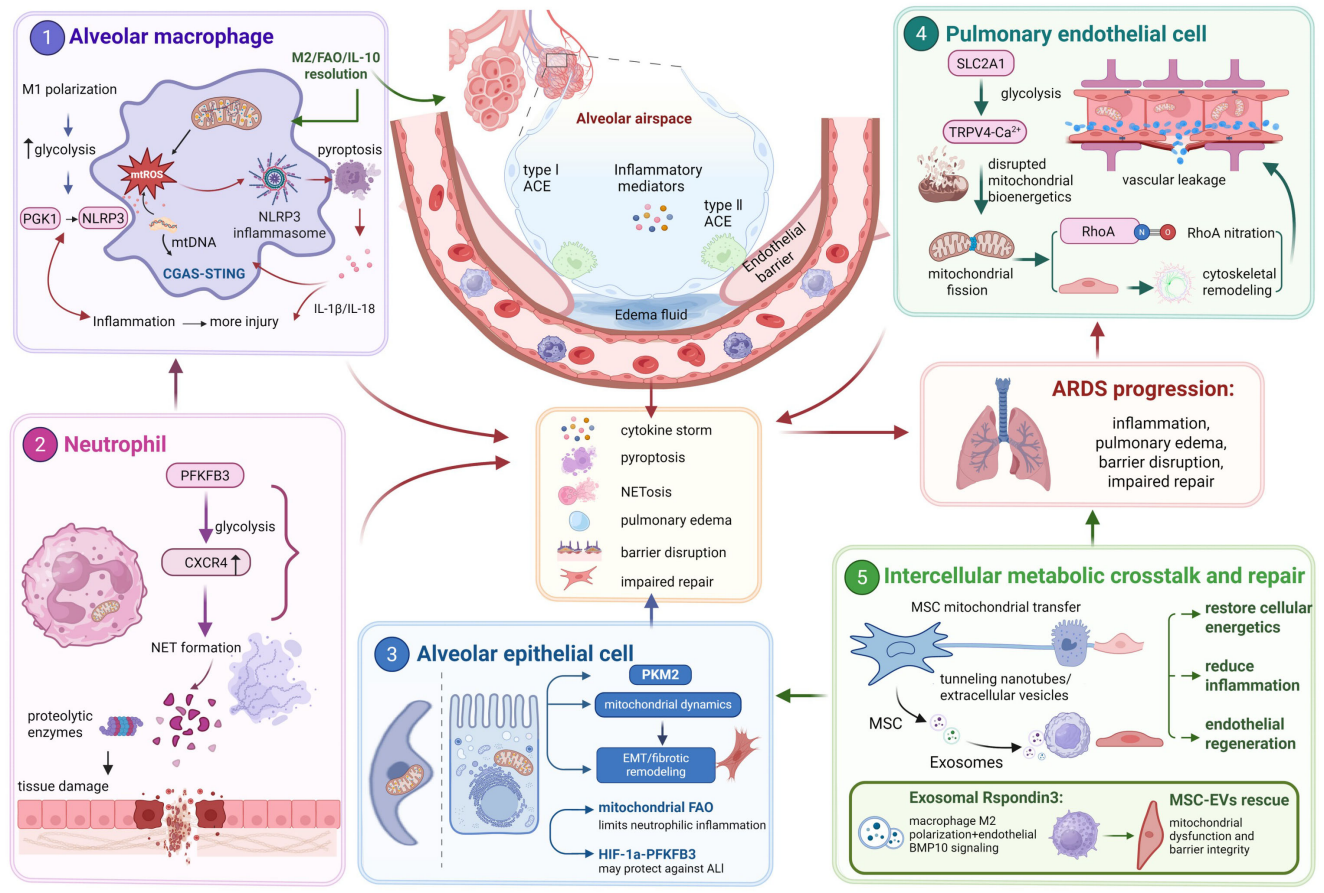
Cell-type-specific mitochondrial and metabolic mechanisms driving ARDS pathogenesis. (1) Alveolar macrophages: Glycolysis upregulates PGK1 to trigger mtROS/mtDNA-mediated cGAS-STING/NLRP3 inflammasome activation, inducing pyroptosis and pro-inflammatory IL-1β/IL-18 release to suppress anti-inflammatory M2 polarization. (2) Neutrophils: PFKFB3-dependent glycolysis activates CXCR4 to promote NETosis and proteolytic tissue injury. (3) Alveolar epithelial cells: PKM2 modulates mitochondrial dynamics, EMT/fibrosis, FAO and HIF-1α-PFKFB3 signaling to regulate lung inflammation. (4) Pulmonary endothelial cells: SLC2A1-glycolysis and TRPV4-Ca²^+^ trigger mitochondrial fission, RhoA nitration, cytoskeletal remodeling and vascular leakage. (5) MSC-mediated intercellular metabolic crosstalk: Mitochondrial transfer and exosomal Rspondin3-containing EVs restore bioenergetics, alleviate inflammation, and repair endothelial barrier to reverse ARDS. Collectively, cell-specific metabolic and mitochondrial dysfunction induces cytokine storm, pyroptosis, barrier disruption and impaired tissue repair, aggravating ARDS progression.

The mechanisms of mitochondrial dysfunction described above, including oxidative stress, impaired mitophagy, aberrant dynamics, reduced biogenesis, mtDNA-mediated inflammation, and metabolic reprogramming, are not uniform across all pulmonary cells. Rather, mitochondrial dysfunction manifests in a cell-type-specific manner across the lung’s diverse cellular landscape. Understanding these distinct cellular contributions not only refines our mechanistic understanding but also informs cell-selective therapeutic strategies. The cell-type-specific mitochondrial alterations in alveolar macrophages, neutrophils, alveolar epithelial cells, and pulmonary endothelial cells are summarized in **Figure 4**. The following subsections discuss each cell type in detail.

### 3.1 Alveolar macrophages

Alveolar macrophages serve as the first line of defense in the lung and play a central role in initiating and resolving inflammation during ARDS (48). Upon activation, these highly plastic cells undergo profound metabolic and mitochondrial reprogramming, shifting toward M1 polarization characterized by robust pro**-**inflammatory cytokine production (18). The hallmark of mitochondrial dysfunction in alveolar macrophages is the convergence of mtROS overproduction and mtDNA release on the NLRP3 inflammasome and cGAS-STING pathways (1), culminating in pyroptosis, a pro-inflammatory form of cell death that amplifies lung injury through the release of abundant DAMPs into the alveolar space (12). This pyroptotic cascade is further amplified by glycolytic reprogramming: key enzymes such as PGK1 actively promote M1 polarization and NLRP3 activation, creating a metabolic circuit that sustains the inflammatory response (18). Thus, in alveolar macrophages, mitochondrial dysfunction primarily perpetuates a feed-forward loop of inflammatory cell death, rather than merely causing metabolic insufficiency, with the mtDNA-cGAS-STING axis operating as a central conduit of this process. As detailed below, this pyroptotic machinery in macrophages stands in stark contrast to the effector programs of other pulmonary cell types, with the mtDNA-cGAS-STING axis representing a cell-type-selective vulnerability that can be therapeutically exploited.

### 3.2 Neutrophils

Neutrophils are among the first immune cells recruited to the lung during ARDS and contribute significantly to tissue damage through release of proteolytic enzymes and formation of neutrophil extracellular traps (NETs) (49, 50). Glycolytic metabolism plays an essential role in supporting these effector functions. In neutrophils, mitochondrial dysfunction and the accompanying glycolytic shift converge on a distinct effector program: NETosis. Unlike alveolar macrophages, which undergo pyroptosis as a consequence of NLRP3/cGAS-STING activation, activated neutrophils extrude NETs, web-like structures composed of decondensed chromatin and granular proteins that mediate pathogen clearance but also cause collateral tissue damage (49). The glycolytic enzyme PFKFB3 has been identified as a critical driver of this process, specifically within the CXCR4hi neutrophil subset. Thus, the glycolysis–mitochondria axis in neutrophils drives NETosis, a cell-type-specific outcome that relies on glycolytic ATP production to fuel the respiratory burst, underscoring that aerobic glycolysis yields fundamentally distinct effector outputs across immune cell populations. This mechanistic divergence, pyroptosis in macrophages versus NETosis in neutrophils, dictates that therapeutic strategies must be cell-type-selective: targeting the cGAS-STING/NLRP3 axis in macrophages versus PFKFB3-driven glycolysis in neutrophils.

### 3.3 Alveolar epithelial cells

Alveolar epithelial cells, comprising type I cells for gas exchange and type II cells for surfactant production and repair, occupy a unique position in ARDS pathogenesis: they are both targets of mitochondrial injury and active participants in its resolution. In stark contrast to the pro-inflammatory effector programs of macrophages and neutrophils, the most distinctive feature of mitochondrial dysfunction in these cells is its biphasic nature, manifesting as either acute epithelial barrier disruption or, should the injury persist, fibrotic remodeling (51). During the early phase, mitochondrial stress drives epithelial barrier disruption; when injury persists, PKM2 modulates mitochondrial dynamics to promote epithelial-mesenchymal transition (EMT), a key step toward sepsis-associated pulmonary fibrosis (6). Importantly, alveolar epithelial cells possess cell-autonomous protective mechanisms that are not observed in myeloid cells: HIF-1α-dependent induction of PFKFB3 dampens acute lung injury, a protective role that directly opposes the pro-inflammatory function of PFKFB3 in neutrophils and macrophages (39). Beyond this cell-intrinsic defense, epithelial cells also exert immunomodulatory effects: enhanced mitochondrial fatty acid oxidation in these cells mitigates neutrophilic inflammation (52), indicating that epithelial cells can actively restrain excessive immune responses through metabolic modulation. Collectively, these observations position alveolar epithelial cells not merely as passive targets of mitochondrial injury but as active determinants of disease trajectory, contributing to either fibrotic progression or repair depending on the prevailing metabolic and mitochondrial state.

### 3.4 Pulmonary endothelial cells

Pulmonary endothelial cells form the vascular barrier and play a central role in regulating leukocyte trafficking, vascular permeability, and inflammatory responses during ARDS (53). Unlike epithelial cells, which can activate protective metabolic programs or undergo fibrotic remodeling depending on the context, endothelial cells are exquisitely sensitive to mitochondrial stress because their monolayer integrity is energetically demanding. More importantly, and distinct from the pyroptotic or NETotic cell death programs seen in immune cells, the central pathological event is excessive Drp1-mediated mitochondrial fission, which fragments the mitochondrial network and disrupts local ATP production at cell–cell junctions (12). This fission program is triggered by at least two distinct upstream signals: (i) activation of the mechanosensitive Ca²^+^ channel TRPV4, which disrupts mitochondrial bioenergetics (10); and (ii) RhoA nitration and cytoskeletal remodeling during inflammatory injury (11). Concurrently, SLC2A1-driven glycolytic reprogramming further compromises barrier function by shunting glucose away from oxidative phosphorylation (54). The net consequence, endothelial hyperpermeability and plasma leakage, is a cell-type-specific outcome of mitochondrial dysfunction in this cell type.

### 3.5 Intercellular metabolic crosstalk

The preceding sections have detailed the mitochondrial and metabolic alterations in alveolar macrophages, neutrophils, alveolar epithelial cells, and pulmonary endothelial cells. The key features of these cell-type-specific changes, including the core mitochondrial events, unique functional consequences, critical effector molecules, and available therapeutic strategies, are summarized in **Table 2**.

**Table 2:** Cell-type-specific mitochondrial dysfunction and available therapeutic strategies in ARDS.

Cell type	Key mitochondrial/metabolic alterations	Pathological consequences in ARDS	Available therapeutic strategies	(Refs.)
Alveolar Macrophages	• mtROS↑ → NLRP3 inflammasome • mtDNA→ cGAS/STING • PGK1-driven glycolysis → M1 polarization • Drp1-mediated mitochondrial fission	• cytokine storm (IL-1β, IL-18) • GSDMD pyroptosis • Sustained pro- inflammatory loop	• 1-Octyl itaconate (cGAS/STING/NLRP3) • Baicalein (Drp1) • H-151 (STING) • Platycodin D3 (PKM2–NLRP3 ) • AKT2–PDK1 blockade (trained immunity)	(1, 12, 15, 18, 39, 46)
Neutrophils	• PFKFB3-driven glycolysis↑ → NETosis	• NET formation • Proteolytic tissue damage • Amplified lung inflammation	• PFKFB3 inhibitors (suppress NETs in CXCR4^+^hi neutrophils)	(37)
Alveolar Epithelial Cells (AECs)	• Drp1 fission↑ /fusion↓ • PKM2 drives EMT • HIF1α-PFKFB3 induction (dual role)	• barrier disruption • EMT→ pulmonary fibrosis • Gas exchange/surfactant ↓	• H_2_ (Trx1/Drp1) • Coral calcium hydride (Trx2/Myo19/Drp1) • GDF15 (suppresses HIF-1α/LDHA) • Promote FAO	(4, 6, 31, 39, 52)
Pulmonary Endothelial Cells	• ↑ Drp1 fission (RhoA nitration) • SLC2A1-driven glycolysis • TRPV4 bioenergetics disruption	• Hyperpermeability • Pulmonary edema • Leukocyte trafficking dysregulation	• MSC mitochondrial transfer (TNTs) • MitoQ (Nrf2) • S1PR3 inhibitors (improve OXPHOS)	(10–12, 21, 22, 53, 54, 58)
Intercellular Crosstalk (MSCs as Regulators)	• MSC mitochondria transfer to injured cells • Rspondin3 → M2 polarization/endothelial BMP10 • miR-181a-5p → PTEN/pSTAT5/SOCS1	• Metabolic reprogramming of recipient cells • Immunometabolic repair • Pro/anti-inflammatory balance	• MSC/ EVs-based therapies • CPT1A modulators (FAO → M2 polarization→ IL-10)	(21, 34, 55, 56)

Note: ↑indicates an increase or upregulation; ↓indicates a decrease or downregulation; →indicates a downstream pathological consequence.

While these cell-autonomous alterations define the distinct pathological contributions of each cell type, the pathogenesis of ARDS is further shaped by intercellular metabolic crosstalk, defined as the exchange of metabolites, mitochondria, and signaling molecules between different cell types, which plays an increasingly recognized role in coordinating inflammatory responses and tissue injury (55). MSCs can transfer functional mitochondria to injured cells through tunneling nanotubes or extracellular vesicles, restoring cellular energetics and reducing inflammation (21), a paradigm of intercellular mitochondrial transfer that offers therapeutic potential. Exosomal communication further contributes to immunometabolic repair: exosomal Rspondin3 serves as a master regulator in sepsis, synergistically activating macrophage M2 polarization and endothelial BMP10 signaling (56), while mesenchymal stromal cell extracellular vesicles rescue mitochondrial dysfunction and improve barrier integrity (34). Collectively, metabolic intermediates derived from mitochondrial metabolism can be transferred between cells to influence recipient cell function, and the coordinated metabolic reprogramming across multiple cell types creates a self-sustaining inflammatory microenvironment that perpetuates ARDS pathogenesis. These intercellular mitochondrial transfer mechanisms not only underscore the complexity of ARDS pathogenesis but also provide a mechanistic foundation for MSC-based and extracellular vesicles (EV)-based approaches, which will be discussed in Section 4.

## 4 Therapeutic strategies targeting mitochondrial dysfunction in ARDS

The recognition of mitochondrial dysfunction as a pathogenic driver in ARDS raises the question of whether restoring mitochondrial homeostasis confers therapeutic benefit. Several lines of evidence support this strategy. Mitochondrial dysfunction sits upstream of inflammation, forms a self-sustaining vicious cycle, and is consistently observed across multiple pulmonary cell types. Moreover, mitochondrial status dictates metabolic polarization (M1 vs. M2), and importantly, diverse interventions targeting distinct mitochondrial pathways, including antioxidants, dynamics modulators, mtDNA signaling inhibitors, and mitochondrial transfer, have all shown efficacy in preclinical ARDS models. This convergence suggests that restoring mitochondrial function can simultaneously ameliorate inflammation, barrier injury, and metabolic dysregulation, an effect unattainable by targeting any single downstream pathway. Therefore, mitochondria represent a rational and promising therapeutic node in ARDS. The following subsections review available mitochondria-targeted strategies, categorized by mechanism and developmental stage, with a comprehensive summary in **Table 3**.

**Table 3:** Summary of mitochondria-targeted therapeutic strategies in ARDS.

Therapeutic class	Agent / compound	Primary target	Mechanism of action	Key findings in preclinical models	Clinical status	(Refs.)
Mitochondria-targeted antioxidants	MitoQ	mtROS	Scavenges mtROS → Nrf2 activation; suppresses mitophagy/NLRP3	Attenuates endothelial hyperpermeability; reduces lung injury in EtOH-LPS models	Phase II pilot trial completed (biomarker modulation)	(16, 22, 58)
	MOTS-c	mtROS / Glycolysis	AMPK–HIF-1α–PFKFB3 → glycolysis↑; oxidative stress↓	Ameliorates CPB-induced lung injury	Preclinical	(46)
Modulators of mitochondrial dynamics	Baicalein	Drp1-mediated fission	Inhibits Drp1-dependent mitochondrial fission in macrophages	Suppresses LPS-induced ALI	Preclinical	(15)
	Hydrogen (H_2_)	Drp1-mediated fission	Trx1/Drp1 pathway modulation; preserves epithelial barrier	Alleviates impaired lung epithelial barrier	Preclinical	(4)
	Coral calcium hydride	Drp1-mediated fission	Trx2/Myo19/Drp1 pathway activation → mitochondrial division	Reduces AT-II cell damage	Preclinical	(31)
Inhibitors of mtDNA-mediated inflammation	1-Octyl itaconate	mtDNA-cGAS/STING-NLRP3 axis	Rescues mitochondrial dysfunction; suppresses cGAS/STING and NLRP3 pathways	Ameliorates alveolar macrophage pyroptosis	Preclinical	(1)
	STING inhibitors (e.g., H-151)	mtDNA–cGAS/STING	Blocks STING → Drp1/N-GSDMD → mtDNA release and pyroptosis	Alleviates sepsis-induced lung injury	Preclinical	(12)
Glycolytic enzyme inhibitors	PFKFB3 inhibitors	PFKFB3 (glycolytic flux)	Reduces NETosis; modulates metabolic reprogramming in neutrophils	Attenuates NET formation and lung injury	Preclinical	(37)
	PKM2 inhibitors	PKM2-NLRP3 axis	Suppresses NLRP3 inflammasome; modulates EMT	Protects against sepsis-associated ALI	Preclinical	(40)
	PDK4 inhibitors	PDK4-lactylation	Reduces lactate accumulation and LPCAT2 lactylation	Modulates sepsis-induced ALI	Preclinical	(41)
Natural compounds	Scutellarin	HIF-1α-glycolysis	Suppresses HIF-1α-mediated Warburg effect → inhibits pyroptosis	Reduces macrophage pyroptosis	Preclinical	(43)
	Shikonin	MCU-mitochondrial Ca²^+^	Modulates MCU-mediated Ca²^+^ → macrophage polarization	Ameliorates LPS-induced ALI	Preclinical	(62)
	Melatonin	Mitophagy / quality control	Enhances mitochondrial quality control; antioxidant	Positive effects in coronavirus infections; potential for ARDS	Approved (other indications); preclinical for ARDS	(60)
Cell-based and EV therapies	MSCs / MSC-EVs	Mitochondrial transfer	Transfers functional mitochondria via TNTs/EVs → bioenergetics restoration; vascular regeneration; macrophage reprogramming	Improves barrier integrity; reduces inflammation in ARDS models	Phase I/II trials completed; safety confirmed	(21, 34, 63–65)
Other approaches	S1PR3 inhibitors (e.g., TY-52156)	Mitochondrial OXPHOS	Improves OXPHOS; inhibits NF-κB	Protects against LPS-induced ARDS	Preclinical	(11)
	17β-Estradiol	Mitochondrial biogenesis	PGC-1α-mediated biogenesis↑	Ameliorates LPS-induced ALI	Preclinical	(14)
	GDF15	HIF-1α–LDHA pathway	Suppresses HIF-1α/LDHA → glycolysis↓	Attenuates sepsis-induced ALI	Preclinical	(42)
	CPT1A modulators	FAO–M2 polarization	FAO↑ → M2 polarization → IL-10↑	Ameliorates ALI	Preclinical	(44)

Note: ↑indicates an increase or upregulation; ↓indicates a decrease or downregulation; →indicates a downstream pathological consequence.

### 4.1 Small molecule modulators

Mitochondria-targeted antioxidants, particularly MitoQ, a TPP-conjugated ubiquinone derivative, have demonstrated therapeutic efficacy in ARDS. MitoQ protects against endothelial barrier hyperpermeability via an Nrf2-dependent mechanism (22) and ameliorates EtOH-LPS induced lung injury by inhibiting mitophagy and NLRP3 inflammasome activation (16). These dual actions highlight the potential of targeting mitochondrial oxidative stress as a therapeutic strategy.

Modulating mitochondrial dynamics also offers therapeutic promise. Drp1 inhibitors, by limiting excessive mitochondrial fission, preserve mitochondrial function and reduce inflammation (5, 15). Complementing this approach, selective inhibition of JNK localized to mitochondria protects against mitochondrial dysfunction and cell death induced by endoplasmic reticulum stress in LPS-induced ALI/ARDS (57), providing an alternative strategy to mitigate mitochondrial stress responses.

Additionally, compounds that enhance mitochondrial bioenergetics and biogenesis have shown efficacy. S1PR3 inhibition protects against LPS-induced ARDS by suppressing NF-κB and improving mitochondrial oxidative phosphorylation (11), while 17β-estradiol ameliorates lung injury by promoting mitochondrial biogenesis and function (14). Among mitochondria-targeted small molecules, MitoQ has advanced the furthest toward clinical application. A pilot randomized trial in patients with septic shock demonstrated that MitoQ significantly improved oxidative stress biomarkers and reduced vasopressor requirements, with a trend toward lower 28-day mortality that did not reach statistical significance (58). In parallel, a Phase 1 trial of low-dose inhaled carbon monoxide in sepsis-induced ARDS patients demonstrated safety and feasibility, with a significant reduction in circulating mtDNA levels in treated patients compared to placebo, although the limited sample size precluded definitive efficacy assessment (59). These early clinical experiences underscore both the promise and the substantial translational gap confronting mitochondria-targeted small molecules, highlighting the urgent need for optimized target specificity, refined dosing regimens, and improved patient stratification in future trials. Collectively, these small molecule modulators target distinct aspects of mitochondrial homeostasis, offering multiple avenues for therapeutic intervention.

### 4.2 Natural compounds

Natural products have gained increasing attention for their ability to modulate mitochondrial function through diverse mechanisms. 1-Octyl itaconate, a derivative of the immunometabolite itaconate, ameliorates alveolar macrophage pyroptosis by rescuing mitochondrial dysfunction and suppressing the cGAS/STING pathway (1); it also activates Nrf2 and modulates metabolic reprogramming, positioning it as a promising multi-target therapeutic. Despite these encouraging preclinical data, itaconate derivatives remain exclusively in the preclinical stage, with no registered clinical trials for ARDS to date. Major barriers include rapid metabolic clearance, limited target tissue accumulation, and the need for medicinal chemistry optimization to improve drug-like properties.

Several natural compounds target mitochondrial dynamics. Baicalein, a flavonoid from Scutellaria baicalensis, suppresses LPS-induced acute lung injury by regulating Drp1-dependent mitochondrial fission in macrophages (15). Hydrogen alleviates impaired lung epithelial barrier by inhibiting Drp1-mediated fission via the Trx1 pathway (4), while coral calcium hydride promotes peripheral mitochondrial division through activation of the Trx2/Myo19/Drp1 pathway (31). These compounds collectively restore the balance of mitochondrial dynamics. While these natural compounds demonstrate mechanistic promise in modulating mitochondrial dynamics, they remain at the preclinical stage for ARDS. Key translational hurdles include poor oral bioavailability, lack of standardized formulations, and the absence of pharmacokinetic data in relevant large-animal models, all of which must be addressed before clinical evaluation can proceed.

Other natural products exert protective effects through distinct mechanisms. Melatonin positively influences mitochondrial quality control during coronavirus infections (60); onion-derived mitochondrial components reprogram macrophage mitochondrial function to limit excessive inflammatory injury (61); scutellarin suppresses HIF-1α-mediated Warburg effect and pyroptosis (43); and shikonin modulates MCU-mediated mitochondrial Ca²^+^ flux and macrophage polarization (62). Together, these diverse mechanisms underscore the therapeutic potential of natural compounds in targeting mitochondrial dysfunction in ARDS.

### 4.3 Cell-based and extracellular vesicle therapies

MSCs have emerged as a promising therapeutic approach for ARDS, primarily through their capacity for mitochondrial transfer. MSCs can transfer functional mitochondria to injured cells via tunneling nanotubes or extracellular vesicles, restoring cellular energetics, reducing inflammation, and promoting vascular regeneration (21, 63).

Mesenchymal stromal cells represent the most clinically advanced mitochondria-targeted strategy for ARDS. Several early-phase trials have confirmed a favorable safety profile, with no significant infusion-related toxicity or serious adverse events observed even at higher doses or following multiple administrations. Trials such as REMODEL and REMEDY have demonstrated improved surrogate endpoints, including diminished inflammatory biomarkers and enhanced markers of endothelial and epithelial repair (64). However, while these studies provide proof-of-concept support, they have not conclusively demonstrated a significant reduction in mortality among ARDS patients, and meta-analyses have revealed inconsistent results across trials (65). Major limitations contributing to this inconsistency include variability in cell potency across manufacturing batches, poor engraftment at target sites, and challenges in large-scale standardization.

MSC-derived extracellular vesicles offer a promising “cell-free” alternative that may overcome some safety and standardization concerns associated with live-cell therapy. Building on this concept, MSC-derived EVs offer an alternative strategy with improved safety profiles. These vesicles rescue mitochondrial dysfunction and improve barrier integrity in clinically relevant ARDS models (34), containing functional mitochondria or mitochondrial components that can be delivered to injured cells without the risks associated with whole-cell therapies.

### 4.4 Other therapeutic approaches

Beyond these major categories of mitochondria-targeted interventions, several additional strategies have shown promise in preclinical studies. Enhancing epithelial mitochondrial fatty acid oxidation mitigates neutrophilic inflammation (52), while targeting pyruvate kinase M2 (PKM2) modulates mitochondrial dynamics and epithelial-mesenchymal transition (EMT) in alveolar epithelial cells (8). Additional compounds, including EGCG (66), Pudilan Xiaoyan (67), GDF15 (42), CPT1A-IL-10 axis modulators (44), and agents that induce trained immunity via AKT2-PDK1-mediated metabolic reprogramming (47), further expand the therapeutic landscape. These diverse approaches collectively underscore the potential of targeting mitochondrial dysfunction as a cornerstone of ARDS treatment.

### 4.5 Mitochondrial extracellular vesicles: emerging diagnostic and therapeutic avenue

Beyond their role as therapeutic vehicles (discussed in Section 4.3), mitochondrial extracellular vesicles (mitoEVs) have recently emerged as a novel class of diagnostic biomarkers and engineered delivery platforms with significant implications for ARDS (68). These vesicles encapsulate mitochondrial components, including mtDNA, proteins, and lipids, and are released into circulation and bronchoalveolar lavage fluid in proportion to the extent of pulmonary mitochondrial injury (34, 69).

Given that mitochondrial dysfunction is a hallmark of ARDS, circulating mitoEVs or those present in bronchoalveolar lavage fluid may serve as valuable diagnostic biomarkers. This potential is supported by the correlation between whole-blood mtDNA copy number and ARDS prognosis (26). From a therapeutic perspective, engineering mitoEVs to deliver functional mitochondrial components to injured cells represents a promising strategy. This approach offers distinct advantages over whole-cell therapies, including reduced immunogenicity and improved safety profiles, positioning mitoEVs as a next-generation platform for mitochondria-targeted intervention in ARDS. However, the clinical translation of mitoEV-based diagnostics and engineered delivery systems faces distinct challenges, including isolation standardization, cargo loading efficiency, and target specificity, which merit further investigation (see Section 5).

## 5 Challenges and future perspectives

### 5.1 Challenges

Despite promising preclinical evidence, several major challenges impede the clinical translation of mitochondria-targeted therapies for ARDS.

Mitochondrial dysfunction manifests in a cell-type-specific manner during ARDS, with alveolar macrophages, neutrophils, endothelial cells, and epithelial cells exhibiting distinct mitochondrial alterations. Single-cell multi-omics approaches, such as those revealing SLC2A1-driven glycolytic reprogramming in pulmonary endothelial cells (54), provide valuable tools to dissect these signatures. Developing cell-selective targeting strategies is therefore critical for achieving therapeutic efficacy while minimizing off-target effects.

Safety considerations also pose substantial hurdles. Mitochondria-targeted therapies, particularly those employing cationic molecules such as TPP, may accumulate in non-target tissues and cause toxicity, necessitating careful long-term safety evaluation. Moreover, modulating fundamental cellular processes, including mitophagy and mitochondrial dynamics, may have unintended consequences that must be thoroughly addressed in preclinical studies.

Translational barriers further complicate clinical development. Most mechanistic evidence derives from ALI models (e.g., LPS- or cecal ligation and puncture-induced lung injury in healthy young animals) rather than clinical ARDS, and these models poorly recapitulate human age, comorbidities, or polypharmacy. Compounding this, validated biomarkers for patient stratification and target engagement are lacking; whole-blood mtDNA copy number and circulating mitoEVs await validation, and optimal biomarker combinations remain undefined. Without reliable biomarkers, promising candidates risk failure in unselected ARDS populations, as seen in prior anti-inflammatory trials.

### 5.2 Future directions

Future research should prioritize the following directions: first, elucidating cell-type-specific mechanisms via single-cell multi-omics and spatial transcriptomics; second, developing more selective mitochondrial targeting strategies with improved safety profiles and tissue specificity; third, identifying robust biomarkers for patient stratification and treatment response monitoring, including circulating mitoEVs and mtDNA; fourth, advancing promising candidates, such as 1-octyl itaconate (1), MitoQ (16, 22), and MSC-derived EVs (34), into well-designed clinical trials; fifth, exploring mitochondrial gene editing technologies, such as DdCBE mediated mtDNA correction, for ARDS prevention and treatment; and finally, investigating mitochondrial replacement therapy as a potential therapeutic avenue. Addressing these priorities will be critical for translating preclinical promise into clinical reality for ARDS management.

## 6 Conclusion

Mitochondrial dysfunction functions as an integrative hub in ARDS pathogenesis, driving a self-sustaining network of inflammation, barrier disruption, and metabolic failure. Importantly, these mitochondrial alterations exhibit cell-type-specific patterns across alveolar macrophages, neutrophils, epithelial cells, and endothelial cells, collectively shaping the pulmonary inflammatory milieu. Targeting mitochondrial homeostasis through antioxidant strategies, modulation of mitophagy and dynamics, restoration of biogenesis, and inhibition of mtDNA-mediated inflammatory signaling represents a promising therapeutic direction. A range of small molecule modulators, natural compounds, and cell-based therapies have shown encouraging results in preclinical models by targeting distinct aspects of mitochondrial homeostasis, including antioxidant defense, dynamics, mtDNA-driven inflammation, and mitochondrial transfer. This therapeutic convergence underscores the rationale for targeting mitochondrial homeostasis as a transformative approach distinct from strategies that neutralize single downstream cytokines or permeability factors. Future research should focus on developing selective targeting strategies, identifying biomarkers, and advancing promising candidates into clinical trials. Mitochondrial-targeted therapies hold the potential to transform ARDS treatment by addressing fundamental mechanisms driving lung injury and inflammation.

## References

[1] Wu YT Xu WT Zheng L Wang S Wei J Liu MY . 4-octyl itaconate ameliorates alveolar macrophage pyroptosis against ARDS via rescuing mitochondrial dysfunction and suppressing the cGAS/STING pathway. Int Immunopharmacol. (2023) 118:110104. doi: 10.1016/j.intimp.2023.110104 37004345

[2] Mokart D Chow-Chine L Sannini A Bisbal M Faucher M . Driving pressure and postoperative ARDS: an intraoperative silent alarm? Intensive Care Med. (2026) 52:1395–6. doi: 10.1007/s00134-026-08457-8 42081103

[3] Xian H Watari K Sanchez-Lopez E Offenberger J Onyuru J Sampath H . Oxidized DNA fragments exit mitochondria via mPTP- and VDAC-dependent channels to activate NLRP3 inflammasome and interferon signaling. Immunity. (2022) 55:1370–85:e8. doi: 10.1016/j.immuni.2022.06.007 35835107 PMC9378606

[4] Long Y Ang Y Chen W Wang Y Shi M Hu F . Hydrogen alleviates impaired lung epithelial barrier in acute respiratory distress syndrome via inhibiting Drp1-mediated mitochondrial fission through the Trx1 pathway. Free Radic Biol Med. (2024) 218:132–48. doi: 10.1016/j.freeradbiomed.2024.03.022 38554812

[5] Jacob S Kosaka Y Bhatlekar S Denorme F Benzon H Moody A . Mitofusin-2 regulates platelet mitochondria and function. Circ Res. (2024) 134:143–61. doi: 10.1161/circresaha.123.322914 38156445 PMC10872864

[6] Feng J Huang X Peng Y Yang W Yang X Tang R . Pyruvate kinase M2 modulates mitochondrial dynamics and EMT in alveolar epithelial cells during sepsis-associated pulmonary fibrosis. J Transl Med. (2025) 23:205. doi: 10.1186/s12967-025-06199-7 39972351 PMC11837412

[7] Hoffman KR Diehl A Nixon P Yong SA Fan E Hodgson C . The six phases of prolonged veno-venous extracorporeal membrane oxygenator support: a conceptual framework. Crit Care. (2026) 30:314. doi: 10.1186/s13054-026-06065-y 42063143 PMC13277115

[8] Lu Q Zemskov EA Sun X Wang H Yegambaram M Wu X . Activation of the mechanosensitive Ca(2+) channel TRPV4 induces endothelial barrier permeability via the disruption of mitochondrial bioenergetics. Redox Biol. (2021) 38:101785. doi: 10.1016/j.redox.2020.101785 33221570 PMC7691184

[9] Thille AW Esteban A Fernandez-Segoviano P Rodriguez JM Aramburu JA Vargas-Errazuriz P . Chronology of histological lesions in acute respiratory distress syndrome with diffuse alveolar damage: a prospective cohort study of clinical autopsies. Lancet Respir Med. (2013) 1:395–401. doi: 10.1016/s2213-2600(13)70053-5 24429204

[10] Peng J Tang R He J Yu Q Wang D Qi D . S1PR3 inhibition protects against LPS-induced ARDS by inhibiting NF-kappaB and improving mitochondrial oxidative phosphorylation. J Transl Med. (2024) 22:535. doi: 10.1186/s12967-024-05220-9 38840216 PMC11151509

[11] Pokharel MD Fu P Garcia-Flores A Yegambaram M Lu Q Sun X . Inflammatory lung injury is associated with endothelial cell mitochondrial fission and requires the nitration of RhoA and cytoskeletal remodeling. Free Radic Biol Med. (2024) 221:125–35. doi: 10.1016/j.freeradbiomed.2024.05.019 38734269 PMC11179967

[12] Zou S Zuo Y Chen Y Zhang T Fu T Li G . Inhibition of STING-induced mitochondrial Drp1/N-GSDMD-mediated MtDNA release alleviates sepsis-induced lung injury. Cell Mol Life Sci. (2025) 82:305. doi: 10.1007/s00018-025-05774-x 40779242 PMC12334783

[13] Zhao H Li X Wang H Li Z Wang Q Zhang Y . A viscous DES-AAV-Foxo1 delivery system with high transfection efficiency for the treatment of corneal endothelial dysfunction by restoring mitochondria-ER contacts. Adv Sci (Weinh). (2026):e75464. doi: 10.1002/advs.75464 42070230 PMC13335468

[14] Wang S Yang H Yang Z Wang Z Li Z . 17beta-estradiol ameliorates LPS-induced acute lung injury via mediating mitochondrial biogenesis and function in rats. Int Immunopharmacol. (2025) 161:114992. doi: 10.1016/j.intimp.2025.114992 40472778

[15] Jiang C Zhang J Xie H Guan H Li R Chen C . Baicalein suppresses lipopolysaccharide-induced acute lung injury by regulating Drp1-dependent mitochondrial fission of macrophages. BioMed Pharmacother. (2022) 145:112408. doi: 10.1016/j.biopha.2021.112408 34801855

[16] Sang W Chen S Lin L Wang N Kong X Ye J . Antioxidant mitoquinone ameliorates EtOH-LPS induced lung injury by inhibiting mitophagy and NLRP3 inflammasome activation. Front Immunol. (2022) 13:973108. doi: 10.3389/fimmu.2022.973108 36059543 PMC9436256

[17] Jiang M Yang Y Zhang Y Zhang P Zhang Q Yang T . Covalent targeting GAPDH by Pudilan formula and baicalein suppresses macrophage Warburg effect to alleviate acute lung injury. Phytomedicine. (2026) 151:157727. doi: 10.1016/j.phymed.2025.157727 41506101

[18] Zhu G Yu H Peng T Yang K Xu X Gu W . Glycolytic enzyme PGK1 promotes M1 macrophage polarization and induces pyroptosis of acute lung injury via regulation of NLRP3. Respir Res. (2024) 25:291. doi: 10.1186/s12931-024-02926-8 39080660 PMC11290129

[19] Kongsintaweesuk S Intuyod K Roytrakul S Seubwai W Sangkhamanon S Charoenlappanit S . System-level multi-omics uncover proteomic, peptidomic, and metabolic remodeling in Microcystin-LR exacerbated chronic kidney disease. Ecotoxicol Environ Saf. (2026) 317:120211. doi: 10.1016/j.ecoenv.2026.120211 42085738

[20] Agarwal S Bankar A Heo L Mitra A Chakraborty A Liu L . CES1 deficiency is associated with metabolic reprograming and endothelial dysfunction in pulmonary arterial hypertension. Am J Respir Crit Care Med. (2026) 212:1315–29. doi: 10.1101/2025.04.25.650735 42085193 PMC13253045

[21] Wang J Meng S Chen Y Wang H Hu W Liu S . MSC-mediated mitochondrial transfer promotes metabolic reprograming in endothelial cells and vascular regeneration in ARDS. Redox Rep. (2025) 30:2474897. doi: 10.1080/13510002.2025.2474897 40082392 PMC11912292

[22] Cen M Ouyang W Zhang W Yang L Lin X Dai M . MitoQ protects against hyperpermeability of endothelium barrier in acute lung injury via a Nrf2-dependent mechanism. Redox Biol. (2021) 41:101936. doi: 10.1016/j.redox.2021.101936 33752110 PMC8005834

[23] White A Wang Z Wang X King M Guo C Mantsounga C . NLRP3 inflammasome activation in cigarette smoke priming for Pseudomonas aeruginosa-induced acute lung injury. Redox Biol. (2022) 57:102467. doi: 10.1016/j.redox.2022.102467 36175355 PMC9618465

[24] Dubey S Yu Z Stephens EM Lazrak A Ahmad I Aggarwal S . Oxidative damage to lung mitochondrial DNA is a key contributor to the development of chemical lung injury. Redox Biol. (2025) 82:103624. doi: 10.1016/j.redox.2025.103624 40209617 PMC12013491

[25] Weinberg SE Chandel NS . Mitochondria reactive oxygen species signaling-dependent immune responses in macrophages and T cells. Immunity. (2025) 58:1904–21. doi: 10.1016/j.immuni.2025.07.012 40763730 PMC12371701

[26] Hernandez-Beeftink T Guillen-Guio B Rodriguez-Perez H Marcelino-Rodriguez I Lorenzo-Salazar JM Corrales A . Whole-blood mitochondrial DNA copies are associated with the prognosis of acute respiratory distress syndrome after sepsis. Front Immunol. (2021) 12:737369. doi: 10.3389/fimmu.2021.737369 34557198 PMC8453061

[27] Li N Liu B Xiong R Li G Wang B Geng Q . HDAC3 deficiency protects against acute lung injury by maintaining epithelial barrier integrity through preserving mitochondrial quality control. Redox Biol. (2023) 63:102746. doi: 10.1016/j.redox.2023.102746 37244125 PMC10199751

[28] Gao F Li Z Peng T Lin B Wang X Dai X . SIRT3-mediated mitophagy by deacetylating ATP5F1A involved in the protective effects of SIGMAR1/Sigma-1 receptor against ferroptosis and microvascular hyperpermeability in lipopolysaccharide-induced acute lung injury. Autophagy. (2026) 22:1221–1235. doi: 10.1080/15548627.2026.2629294 41655128 PMC13185463

[29] Liu Y Ye P Tang C Jiang M Shen Y Wang X . CDDO-imidazolide ameliorates sepsis-induced ARDS by enhancing mitophagy via the Nrf2 pathway to prohibit alveolar macrophage pyroptosis and HMGB1 release. Acta Biochim Biophys Sin (Shanghai). (2025) 57:1864–74. doi: 10.3724/abbs.2025092 40599023 PMC12666672

[30] Yu J Lu Z Chen B He X Zhao W Cao H . Liang-Ge-San protects against viral infection-induced acute lung injury through inhibiting alpha7nAChR-mediated mitophagy. Phytomedicine. (2024) 135:156231. doi: 10.1016/j.phymed.2024.156231 39566410

[31] Li Q Ang Y Zhou QQ Shi M Chen W Wang Y . Coral calcium hydride promotes peripheral mitochondrial division and reduces AT-II cells damage in ARDS via activation of the Trx2/Myo19/Drp1 pathway. J Pharm Anal. (2025) 15:101039. doi: 10.1016/j.jpha.2024.101039 40177064 PMC11964661

[32] Tian S Zhang Y Liu C Zhang H Lu Q Zhao Y . Double-edged mitophagy: balancing inflammation and resolution in lung disease. Clin Sci (Lond). (2025) 139:1047–72. doi: 10.1042/cs20256705 41026942 PMC12599259

[33] Li H Dai X Zhou J Wang Y Zhang S Guo J . Mitochondrial dynamics in pulmonary disease: Implications for the potential therapeutics. J Cell Physiol. (2024) 239:e31370. doi: 10.1002/jcp.31370 38988059

[34] Dutra Silva J Su Y Calfee CS Delucchi KL Weiss D McAuley DF . Mesenchymal stromal cell extracellular vesicles rescue mitochondrial dysfunction and improve barrier integrity in clinically relevant models of ARDS. Eur Respir J. (2021) 58:2002978. doi: 10.1183/13993003.02978-2020 33334945 PMC8318599

[35] Yuan ZC Zeng N Liu L Wang T Dai LQ Wang H . Mitochondrial damage-associated molecular patterns exacerbate lung fluid imbalance via the formyl peptide receptor-1 signaling pathway in acute lung injury. Crit Care Med. (2021) 49:e53–62. doi: 10.1097/ccm.0000000000004732 33165026

[36] Luo L Zhuang X Fu L Dong Z Yi S Wang K . The role of the interplay between macrophage glycolytic reprogramming and NLRP3 inflammasome activation in acute lung injury/acute respiratory distress syndrome. Clin Transl Med. (2024) 14:e70098. doi: 10.1002/ctm2.70098 39623879 PMC11612265

[37] Liu D Xiao M Zhou J Wang P Peng J Mao W . PFKFB3 promotes sepsis-induced acute lung injury by enhancing NET formation by CXCR4(hi) neutrophils. Int Immunopharmacol. (2023) 123:110737. doi: 10.1016/j.intimp.2023.110737 37543012

[38] Yuan D Yang F Hou L Zhang Y Pang X Du Y . PFKFB2-driven glycolysis promotes dendritic cell maturation and exacerbates acute lung injury. Adv Sci (Weinh). (2025) 12:e02428. doi: 10.1002/advs.202502428 40736063 PMC12499423

[39] Vohwinkel CU Burns N Coit E Yuan X Vladar EK Sul C . HIF1A-dependent induction of alveolar epithelial PFKFB3 dampens acute lung injury. JCI Insight. (2022) 7:e157855. doi: 10.1172/jci.insight.157855 36326834 PMC9869967

[40] Wang J Yao L Cui K Zhao J Yang M . Platycodin D3 protects against sepsis-associated acute lung injury through direct inhibition of the PKM2-NLRP3 axis. Cell Signal. (2026) 142:112443. doi: 10.1016/j.cellsig.2026.112443 41748044

[41] Deng Y Qiu Y Li X Gong T Guo J Liang H . PDK4-driven lactate accumulation facilitates LPCAT2 lactylation to exacerbate sepsis-induced acute lung injury. Cell Death Differ. (2026) 33:557–73. doi: 10.1038/s41418-025-01585-6 41057687 PMC13035903

[42] Kuang X Niu Z Huang Z Cai X Wang L Zhang Y . GDF15 attenuates sepsis-induced acute lung injury by suppressing the HIF-1alpha/LDHA pathway. Int Immunopharmacol. (2025) 163:115198. doi: 10.1016/j.intimp.2025.115198 40669249

[43] Wu J Liu F Shen J Zhang H Liu Y Sun J . Scutellarin suppresses Mycobacterium tuberculosis-induced pyroptosis in macrophages by inhibiting the HIF-1alpha-mediated Warburg effect. Redox Rep. (2025) 30:2565861. doi: 10.1080/13510002.2025.2565861 41051976 PMC12502121

[44] Wang M Wu D Liao X Hu H Gao J Meng L . CPT1A-IL-10-mediated macrophage metabolic and phenotypic alterations ameliorate acute lung injury. Clin Transl Med. (2024) 14:e1785. doi: 10.1002/ctm2.1785 39090662 PMC11294017

[45] Sang SY Wang YJ Liang T Liu Y Liu JJ Li H . Protein 4.1R regulates M1 macrophages polarization via glycolysis, alleviating sepsis-induced liver injury in mice. Int Immunopharmacol. (2024) 128:111546. doi: 10.2139/ssrn.4635106 38237224

[46] Shen Z Lu P Jin W Wen Z Qi Y Li X . MOTS-c promotes glycolysis via AMPK-HIF-1alpha-PFKFB3 pathway to ameliorate cardiopulmonary bypass-induced lung injury. Am J Respir Cell Mol Biol. (2025) 73:353–68. doi: 10.1165/rcmb.2024-0533oc 40035775

[47] Sun Z Meng H Wang X Guo X He W Zhou Y . Trained immunity attenuated acute lung injury by activating alveolar macrophages via AKT2-PDK1 axis-mediated metabolic reprogramming. J Transl Med. (2025) 23:1412. doi: 10.1186/s12967-025-06879-4 41437043 PMC12723939

[48] Hu S Wang Y Zhou W Zhu L Chen Y Li Q . Exosomes derived from bone marrow mesenchymal stem cells alleviate sepsis-induced ARDS via inhibition of HOXA9-mediated glycolysis in alveolar macrophages. Respir Res. (2026) 27:181. doi: 10.1186/s12931-026-03564-y 41845452 PMC13112714

[49] Li J Wang Y Liu J Wang N Shen P Zhou W . Cannabidiol hinders lipopolysaccharide-induced neutrophils migration to the lungs through suppressing nuclear factor kappa-B signal and expression of interleukin-1 beta in macrophages. Phytomedicine. (2026) 155:158122. doi: 10.1016/j.phymed.2026.158122 41935460

[50] Luo Y Li M Lin S Gong Z Wang S Zhou Z . Fndc5 modification optimizes the therapeutic effect of rat MSCs on sepsis-induced ALI/ARDS via activating the PI3K/AKT signaling pathway. Stem Cell Res Ther. (2026) 17:100. doi: 10.1186/s13287-026-04903-y 41580845 PMC12977885

[51] Zhou H Liang C Cheng F Wang K Zhang Y Chen J . Mitochondria-targeting polymeric micelles for intervertebral disc degeneration alleviation via coordinated cascade energetic intervention. ACS Nano. (2025) 19:41121–35. doi: 10.1021/acsnano.5c13916 41289568

[52] Chung KP Cheng CN Chen YJ Hsu CL Huang YL Hsieh MS . Alveolar epithelial cells mitigate neutrophilic inflammation in lung injury through regulating mitochondrial fatty acid oxidation. Nat Commun. (2024) 15:7241. doi: 10.1038/s41467-024-51683-1 39174557 PMC11341863

[53] Chan WC Kwok ML Qu X Abdelkarim H Le J Yang D . Therapeutic targeting of endothelial calcium signaling accelerates the resolution of lung injury. Signal Transduct Target Ther. (2025) 10:375. doi: 10.1038/s41392-025-02461-y 41253746 PMC12627862

[54] Long D Lu Z Fang B Tian Y Fu H Dong Y . Single-cell multi-omics reveals SLC2A1-driven glycolytic reprogramming of pulmonary endothelial cells in sepsis-associated lung injury. Biochim Biophys Acta Mol Basis Dis. (2026) 1872:168050. doi: 10.1016/j.bbadis.2025.168050 40946874

[55] Peng Y Mei S Qi X Tang R Yang W Feng J . PGC-1alpha mediates migrasome secretion accelerating macrophage-myofibroblast transition and contributing to sepsis-associated pulmonary fibrosis. Exp Mol Med. (2025) 57:759–74. doi: 10.1038/s12276-025-01426-z 40164683 PMC12046055

[56] Fang B Long D Li T Fu H Dong Y Tian Y . Exosomal Rspondin3 serves as a master regulator of immunometabolic repair in sepsis: synergistic activation of macrophage M2 polarization and endothelial BMP10 signaling. Int Immunopharmacol. (2026) 176:116372. doi: 10.1016/j.intimp.2026.116372 41839470

[57] Li C Ma D Chen Y Liu W Jin F Bo L . Selective inhibition of JNK located on mitochondria protects against mitochondrial dysfunction and cell death caused by endoplasmic reticulum stress in mice with LPS-induced ALI/ARDS. Int J Mol Med. (2022) 49:85. doi: 10.3892/ijmm.2022.5141 35514298 PMC9106374

[58] Moradbaki H Hassani-Pajooh H Valizade-Hasanloei MA Hatamkhani S Amini K Zamanirafe M . A pilot double-blind, placebo-controlled, randomized clinical trial of MitoQ in the treatment of septic shock: evaluating its effects on clinical outcomes and oxidative stress biomarker. Naunyn Schmiedebergs Arch Pharmacol. (2026) 399:2175–88. doi: 10.1007/s00210-025-04526-9 40820063

[59] Fredenburgh LE Perrella MA Barragan-Bradford D Hess DR Peters E Welty-Wolf KE . A phase I trial of low-dose inhaled carbon monoxide in sepsis-induced ARDS. JCI Insight. (2018) 3:e124039. doi: 10.1172/jci.insight.124039 30518685 PMC6328240

[60] Mehrzadi S Karimi MY Fatemi A Reiter RJ Hosseinzadeh A . SARS-CoV-2 and other coronaviruses negatively influence mitochondrial quality control: beneficial effects of melatonin. Pharmacol Ther. (2021) 224:107825. doi: 10.1016/j.pharmthera.2021.107825 33662449 PMC7919585

[61] Xu Q Teng Y Huang Y Mu J Teng L Qian H . Onion-mitochondria inhibit lipopolysaccharide-induced acute lung injury by shaping lung macrophage mitochondrial function. Adv Sci (Weinh). (2025) 12:e06107. doi: 10.1002/advs.202506107 41051350 PMC12752597

[62] Bao-Yuan H Shu-Ru L Le-Xin C Liang-Liang B Cheng-Cheng L Chun-Qi X . Shikonin ameliorated LPS-induced acute lung injury in mice via modulating MCU-mediated mitochondrial Ca(2+) and macrophage polarization. Phytomedicine. (2024) 135:156043. doi: 10.1016/j.phymed.2024.156043 39366155

[63] Su Y Silva JD Doherty D Simpson DA Weiss DJ Rolandsson-Enes S . Mesenchymal stromal cells-derived extracellular vesicles reprogramme macrophages in ARDS models through the miR-181a-5p-PTEN-pSTAT5-SOCS1 axis. Thorax. (2023) 78:617–30. doi: 10.1136/thoraxjnl-2021-218194 35948417 PMC10314068

[64] Zarrabi M Shahrbaf MA Nouri M Shekari F Hosseini SE Hashemian SR . Allogenic mesenchymal stromal cells and their extracellular vesicles in COVID-19 induced ARDS: a randomized controlled trial. Stem Cell Res Ther. (2023) 14:169. doi: 10.1186/s13287-023-03402-8 37365605 PMC10294333

[65] Wang F Li Y Wang B Li J Peng Z . The safety and efficacy of mesenchymal stromal cells in ARDS: a meta-analysis of randomized controlled trials. Crit Care. (2023) 27:31. doi: 10.1186/s13054-022-04287-4 36670442 PMC9857915

[66] Jin X Weng Q Qian K Liu F . Network pharmacology analysis and experimental validation of EGCG's therapeutic effects against acute lung injury in sepsis. Int Immunopharmacol. (2025) 162:115175. doi: 10.1016/j.intimp.2025.115175 40652647

[67] Xu N Lu X Yu J Gong J Guo H Chen K . Modulation of glycolytic metabolic reprogramming and the TLR4/NF-kappaB/NLRP3 pathway: comparative analysis of anti-inflammatory activity between two dosage forms of Pudilan xiaoyan. J Ethnopharmacol. (2025) 352:120204. doi: 10.1016/j.jep.2025.120204 40571230

[68] Miao R Wang S Yin H Zhu R Yin Y Liao W . Scutellaria baicalensis-derived extracellular vesicles alleviate inflammatory bowel disease by inhibiting the NF-kappaB/NLRP3 pathway. Int J Nanomedicine. (2026) 21:586382. doi: 10.2147/ijn.s586382 42079434 PMC13135100

[69] Liu C Xiang G Cao Y Xu J Hu X Deng S . Smart nebulized ROS-responsive hydrogel microspheres loaded with UC-MSCs-derived exosomes for the treatment of acute lung injury. Mater Today Bio. (2026) 37:102868. doi: 10.1016/j.mtbio.2026.102868 41696152 PMC12905755

